# A Cohort Study Assessing the Impact of Anki as a Spaced Repetition Tool on Academic Performance in Medical School

**DOI:** 10.1007/s40670-023-01826-8

**Published:** 2023-07-01

**Authors:** Michael M. Gilbert, Timothy C. Frommeyer, Garrett V. Brittain, Nickolas A. Stewart, Todd M. Turner, Adrienne Stolfi, Dean Parmelee

**Affiliations:** 1 Boonshoft School of Medicine at Wright State University, Dayton, OH USA; 2 Departments of Pharmacology & Toxicology, and Dermatology, Boonshoft School of Medicine at Wright State University, Dayton, OH USA; 3 Orthopedics, Mount Carmel Health System, Columbus, OH USA

**Keywords:** Spaced repetition, Anki, Retrieval practice, Flashcards, Medical education

## Abstract

**Introduction:**

Anki is an application that capitalizes upon the techniques of spaced repetition and is increasingly utilized by medical students for examination preparation. This study examines the impact of Anki usage in a medical school curriculum on academic performance. Secondary objectives analyzed individual Anki utilization and a qualitative assessment of Anki use.

**Methods:**

A cohort-control study was conducted at Boonshoft School of Medicine. One hundred thirty first-year medical students were enrolled in an Anki utilization training program from July 2021 to September 2021. Training included educational Anki courses and subsequent survey data collection over Anki usage. Data variables included all course final examinations, the Comprehensive Basic Science Exam (CBSE), individual Anki user statistics, nationally standardized exams scores, and Qualtrics surveys on student perceived ease of use.

**Results:**

Seventy-eight students reported using Anki for at least one of the exams, and 52 students did not use Anki for any exam. Anki users scored significantly higher across all four exams: Course I (6.4%; *p* < 0.001); Course II (6.2%; *p* = 0.002); Course III (7.0%; *p* = 0.002); and CBSE (12.9%; *p* = 0.003). Students who reported higher dependency on Anki for studying performed significantly better on the Course I, II, and CBSE exams.

**Conclusion:**

Anki usage may be associated with an increase in standardized examination scores. This supports Anki as an evidence-based spaced repetition and active retrieval learning modality for medical school standardized examinations. There was little correlation between its specific statistical markers and examination performance. This is pertinent to physicians and medical students alike as the learning and preservation of biomedical knowledge is required for examinations and effective clinical care.

## Introduction

Teaching and learning strategies in medical education evolve alongside an ever-expanding body of scientific knowledge. Institutions and learners have favored lecture, assigned reading, quizzes, and summative examinations to drive mastery of content. Now, the digital age has generated new tools to support this mastery such as online interactive platforms [1-3]. Tools and resources that embrace spaced repetition and retrieval-based practice have demonstrated considerable effectiveness and use in all disciplines [4–7]. Spaced repetition derives from the spacing effect coined in Hermann Ebbinghaus’s book, *Memory: A Contribution to Experimental Psychology*, in which he found that continuous repetition spaced in time produces stronger memories [8]. Utilizing retrieval practice, learners can take advantage of two important effects: the spacing effect and the testing effect. The spacing effect suggests that retention is enhanced when information is encountered repeatedly over time, while the testing effect highlights the improvement in performance that comes from actively stimulating memory through testing [9, 10]. By incorporating these principles from cognitive psychology, retrieval practice reinforces memory and enhances the chances of successful recall in the future [11]. Higher education and medical education continue to explore this important psychological construct along with retrieval-based practice [12–15]. Anki™ is one learning tool that medical students and residents are using to improve their mastery of curricular content. It is an open-sourced flashcard application built on the algorithms of spaced repetition, and it incorporates increasing intervals between flashcards requiring active retrieval practice that leads to greater retention and transfer of data to long-term memory.

Although studies have demonstrated that flashcard-based applications tend to improve medical student retention of content [6, 16–18] and there is abundant evidence on the benefit of spaced repetition [12], there is limited research on Anki’s outcomes with this population. One study demonstrated Anki usage significantly correlated with improved US Medical Licensing Exam (USMLE) step 1 performance, where completion of an additional 1500 Anki cards led to only a one-point increase in the score [17]. Lu and colleges also examined USMLE step 1 performance by surveying 2nd, 3rd, and 4th year medical students Anki usage. They found Anki usage was associated with higher USMLE step 1 scores and a higher perceived level of knowledge retention [19]. More recently, Wothe et al. demonstrated in 165 students surveyed, daily Anki use was correlated with increased Step 1 scores, but not Step 2 scores. Interestingly, they found an association between Anki use and increased sleep quality [20] Sun et al., however, did not find benefit of flashcard use in a medical student psychiatry course, but this may have been due to the short 1-month interval of the study [16]. Within residency education, both medicine and surgery subspecialty residents are using Anki for preparation for in-service exams, and studies have demonstrated strong positive correlation with examination scores [21, 22].

There are numerous ways in which Anki may be utilized to support learners mastering a particular subject. These include the specific types and numbers of flashcards used and/or the time intervals between flashcards, as well as whether the flashcards are user-sourced (created by the specific learner community) or homemade (created by an individual). The Anki application provides the learner with continuous data: number of flashcards per day, how many days in a row of completed cards, and percentage of flashcards “passed” of all flashcards completed. The user may supplement the data generation with “add-ons” such as “True retention” that tracks number of reviews completed each week and month, number of reviews with failing scores, and number of new cards mastered. The learner is therefore provided extensive feedback on level of effort and progress toward desired mastery of the subject matter.

This study was conducted at Boonshoft School of Medicine (BSOM) during the 2021–2022 academic year. We examined how Anki usage impacted the academic performance of more than half of a first-year class (78/130) for the year. The study’s primary aim was to analyze the statistics embedded into individual user accounts that track Anki usage and their summative examination data: one in-house final examination; two National Board of Medical Examiners Customized Exams (NBME) administered at the conclusion of each major course (Courses I, II, III); and the Comprehensive Basic Science Subject Exam (CBSE) administered at the end of the year. We also explored how a learner can best use Anki to achieve higher exam performance in medical school and how the regular and consistent use of Anki impacts confidence in preparation for an exam.

## Methods

### Phase 1: “How-to-Use” Module

The Anki system requires an initial time investment to fully learn its capabilities, and students are often reluctant to devote the time and effort to learn how to use it to the level of benefit. For this phase, the study incorporated a “how to use” training program open to all first-year medical students at BSOM (130 of 130 students attended). The program consisted of an hour-long introductory classroom session (overview of the system, why it might be useful to learn, importance of spaced repetition) and a second 2-h-long classroom session (specifics on how to set up the system, general user interface, Anki implementation, finding relevant flashcards, and Anki user statistics). Approximately, 126 of 130 students attended the second session. Four second-year medical students who had extensive Anki knowledge and experience provided all instruction and guidance as well as designed and conducted this study.

In addition, a series of individual and small group guidance/instruction times were offered by the second-year medical students during which the following was provided: detailed instructions on how to optimize the system for specific needs such as “add-ons,” weekly instructions on finding Anki flashcards that matched current curricular topics, and further guidance and troubleshooting throughout the first major course of the year (Course I). During this phase, there was no recording of names of students who attended any component.

## Phase 2: Data Collection and Analysis

In this phase, the entire class of 2025 (*n* = 130) was invited to participate in the study. Those who agreed to participate in phase 2 (both those who attended any or all “how to use” sessions and students that did not attend either) completed an informed consent. Those who had participated in the phase 1 and provided informed consent submitted their Anki decks containing all user statistics to the Office of Medical Education (OME) after each standardized exam. At this time, students additionally completed a brief survey through a standardized email asking about dependency on Anki for studying (low, medium, high), how confident they felt about the exam (not at all, slightly, somewhat, fairly, completely), and how prepared they felt for the exam (not at all, slightly, somewhat, fairly, completely). Consenting student names, submitted Anki statistics, and survey data were matched with Medical College Admissions Test (MCAT) percentiles and NBME exam scores by the OME and then coded and fully deidentified for analyses. MCAT and NBME scores for students who did not use Anki for any course or exam were also collected and deidentified by the OME, and these students served as a control group. MCAT percentiles were included as a study covariate to control for differences in baseline academic performance between Anki users and non-users.

MCAT percentiles, exam scores, and Anki user statistics were summarized with mean (standard deviation, SD) and range, and differences in exam scores between Anki users and non-users were summarized with mean and 95% confidence interval. MCAT percentiles and exam scores were approximately normally distributed and were analyzed with parametric statistics. Most Anki user statistics were not normally distributed, so associations with exam scores were determined with Spearman rank correlation coefficients.

The examinations occurred after each course: Course I, II, and III. Course I is titled Origins consists of molecular and cellular biochemistry, and an introduction to pathology and pharmacology. Course II is titled Host and Defense, and consists of immunology and microbiology. Course III is titled Staying Alive and focuses on cardiovascular, renal, and pulmonary physiology and pathology. The last exam, the CBSE, was administered at the end of the year. For each of the four exams, mean (SD) scores were compared between students who used Anki for that exam and students who did not use Anki for any exam. Students who used Anki at least once, but not for a specific exam, were excluded from the analysis for that exam. The Anki users and non-users were first compared on MCAT percentiles with two-sample *t* tests; differences were statistically significant, so analysis of covariance with MCAT scores as the covariate was used for all comparisons of exam scores. All exams were scored on 0–100% scales, and for course exams, the passing threshold was ≥ 70%.

To determine whether the degree of dependency on Anki for studying, confidence about the exam, or preparation for the exam were associated with exam scores, one-way analysis of variance (ANOVA) was used to compare the exam scores among the different levels of each variable. When the ANOVA was significant, Bonferroni tests with adjustments for multiple comparisons were applied to determine specific differences among the levels. For confidence and preparation levels, responses were collapsed into “not at all/slightly,” “somewhat/fairly,” and “completely” due to small sample sizes for some of the levels.

A total of 21 Anki user statistics were assessed for associations with exam scores using Spearman rank correlation coefficients. Descriptions of each user statistic are provided in Supplementary Table 1. Anki user statistics that were significantly correlated with each exam were then entered into multiple linear regression models with MCAT scores and levels of dependence on Anki, confidence, and preparation to determine independent associations with exam scores. Forward stepwise variable selection was used, with *p* < 0.05 for entry and *p* < 0.10 for remaining in the model. All analyses were conducted with SPSS v.29 (IBM Corporation, Armonk, NY). For all analyses, *p* values less than 0.05 were considered statistically significant. Finally, this research was conducted with approval from the Wright State Institutional Review Board (IRB#7178).

## Results

Of the 130 students from the BSOM Class of 2025, 78 (60.0%) used Anki for at least one exam. Among the Anki users, 48/78 (61.5%) used it for Course I, 39/78 (50.0%) for Course II, 23/78 (29.5%) for Course III, and 10/78 (12.8%) used Anki for the CBSE exam. Students who used Anki for at least one exam had significantly higher mean (SD) MCAT percentiles compared to those who did not use Anki at all [73 (16), *n* = 78 vs. 65 (19), *n* = 52, *p* = 0.005]. When comparing only students who used Anki for a particular course/exam to students who never used Anki, the differences were 73 (16) vs. 65 (19), *p* = 0.021 for Course I; 74 (16) vs. 65 (19), *p* = 0.018 for Course II; 74 (13) vs. 65 (19), *p* = 0.034 for Course III; and 74 (13) vs. 65 (19), *p* = 0.138 for the CBSE.

Table 1 shows the exam scores and comparisons between Anki users and non-users for each course/exam. After taking MCAT scores into account, Anki users scored significantly higher than non-users on all exams. Mean score differences with 95% confidence intervals (95% CI) ranged from 6.2 (95% CI 2.4–9.9) for Course II to 10.7 (95% CI 2.6–18.7) for the CBSE.

**Table 1:** Comparisons of NBME exam scores between Anki users and non-users

Course	Statistic	Anki users	Anki non-users	Difference (95% CI)^a^	*p* value^a^
I	Mean (SD) Range *n*	88.5 (7.2) 68–98 48	80.1 (8.0) 66–97 52	6.4 (3.8–8.9)	< 0.001
II	Mean (SD) Range *n*	85.6 (8.2) 62–97 39	78.1 (9.7) 58–98 52	6.2 (2.4–9.9)	0.002
III	Mean (SD) Range *n*	84.6 (7.1) 68–95 23	76.4 (9.5) 49–95 52	7.0 (2.6–11.4)	0.002
CBSE	Mean (SD) Range *n*	72.2 (13.9) 47.2–100.0 10	59.3 (11.9) 38.9–91.7 52	10.7 (2.6–18.7)	0.011

*CBSE* Comprehensive Basic Science Exam, *CI* confidence interval, *SD* standard deviation. Scores are in percent, and differences are Anki-user scores minus non-user scores

^a^Difference (95% CI) and *p* value are adjusted for the effect of differences in MCAT percentiles using analysis of covariance with MCAT percentile as the covariate

Exam scores and proportions of Anki users at each level of dependency on Anki, confidence for exam, and preparedness for exam are shown in Table 2. Most students had a high dependency on Anki for studying, with proportions ranging from 47.9% for Course I to 70.0% for the CBSE. High-dependency Anki users scored significantly higher than low- or medium-dependency users for Course I, Course II, and the CBSE, but not for Course III. Students who recorded themselves as completely confident had higher exam scores compared to students who reported themselves as not at all confident for Course I and Course III, and students who reported themselves as completely prepared scored higher than students who reported themselves as not at all prepared for Course I and Course II (Table 2).

**Table 2:** Proportions of Anki users and mean exam scores by levels of dependency on Anki, confidence for exam, and preparedness for exam

	Course I		Course II		Course III		CBSE	
Variable and level	*n* (%)	Mean (SD)	*n* (%)	Mean (SD)	*n* (%)	Mean (SD)	*n* (%)	Mean (SD)
Dependency on Anki Low Medium High Total	7 (14.6) 18 (37.5) 23 (47.9) 48 (100)	83.3 (10.5) 85.9 (6.5) 92.0 (4.5)*,** *p* = 0.008	6 (15.4) 9 (23.1) 24 (61.5) 39 (100)	74.5 (8.7) 85.3 (7.7)* 90.9 (3.4)* *p* = 0.001	2 (8.7) 5 (21.7) 16 (69.6) 23 (100)	75.5 (10.6) 85.8 (6.4) 85.4 (6.6) *p* = 0.166	0 (0.0) 3 (30.0) 7 (70.0) 10 (100)	na 59.3 (10.8) 77.8 (11.4) *p* = 0.044
How confident for exam Not at all/slightly Somewhat/fairly Completely Total	2 (4.2) 32 (66.7) 14 (29.2) 48 (100)	87.0 (1.4) 86.7 (7.9) 92.7 (2.8)*** *p* = 0.011	7 (17.9) 24 (61.5) 8 (20.5) 39 (100)	80.7 (12.5) 82.8 (8.8) 87.9 (5.8) *p* = 0.193	3 (13.0) 17 (73.9) 3 (13.0) 23 (100)	73.7 (5.5) 85.4 (5.8)*** 91.0 (3.5)*** *p* = 0.003	5 (50.0) 4 (40.0) 1 (10.0) 10 (100)	66.9 (13.2) 71.9 (6.4) 100 (-) *p* = 0.519
How prepared for exam Not at all/slightly Somewhat/fairly Completely Total	2 (4.2) 38 (79.2) 8 (16.7) 48 (100)	69.5 (2.1) 88.4 (6.2)*** 93.4 (3.0)*** *p* < 0.001	4 (10.3) 25 (64.1) 10 (25.6) 39 (100)	81.1 (10.0) 85.0 (8.0) 91.3 (2.4)*** *p* = 0.005	1 (4.3) 16 (69.6) 6 (26.1) 23 (100)	74.0 (-) 82.8 (6.4) 91.3 (3.4) *p* = 0.006	9 (90.0) 0 (0.0) 1 (10.0) 10 (100)	69.1 (10.5) na 100 (-) na

*CBSE* Comprehensive Basic Science Exam, *NA* not applicable, *SD* standard deviation. Mean (SD) is the score on the course exam

*p* values are from one-way analysis of variance. Superscripted a, b, and c are from Bonferroni multiple comparison tests

* *p* < 0.05 vs. low dependency; ** *p* < 0.05 vs. medium dependency; *** *p* < 0.05 vs. not at all/slightly

Descriptive statistics and their meanings (mean, SD, range, *n*) for all 21 Anki user statistics for each exam are shown in Supplemental Table 1 and Table 2. Ten of the user statistics were correlated with at least one exam and are shown in Table 3. Seven user statistics were correlated with Course I exam score, four with Course II exam score, and two with the CBSE. None of the user statistics were correlated with Course III.

**Table 3:** Correlations between Anki user statistics and exam scores for statistics significantly correlated with at least one exam

	Course I		Course II		Course III		CBSE	
Anki user statistic	*n*	*r*	*n*	*r*	*n*	*r*	*n*	*r*
Days studied	39	0.350*	35	0.300	19	− 0.113	10	0.596
Days learned	42	0.517**	35	0.320	19	0.170	8	0.727*
Daily average	47	0.280	38	0.392*	21	− 0.127	10	0.182
Longest streak	47	0.304*	38	0.133	22	− 0.001	10	0.381
Current streak	47	0.383**	38	0.292	22	0.053	10	0.474
Retention rate	37	0.258	31	0.037	17	0.385	6	− 0.943**
Average Anki time per day (min)	38	0.472**	30	0.171	17	− 0.159	6	0.657
AnKing % mature	38	0.371*	35	0.489**	19	0.264	10	0.237
AnKing % unseen/suspend/new	38	− 0.366*	35	− 0.391*	19	− 0.192	10	− 0.328
Collection # mature	39	0.228	35	0.470**	19	0.159	10	0.055

*CBSE* Comprehensive Basic Science Exam, *r*, Spearman rank correlation coefficient. Anki user statistics that were not correlated with any of the exams were total days, days studied (% of total days), average interval (months), average ease (%), total flashcards, AnKing % young/learn/relearn, collection # young/learn/relearn/buried, collection # suspended/new, mature % correct, young % correct, and learning % correct. A mature card is defined as one that has an interval of 21 days or greater. An unseen/suspended/new card is defined as a card that has not been learned or seen. Retention rate is the percentage of cards correct in the young and mature phase. The collection is the total number of cards in users Anki profile

* *p* < 0.05; ** *p* < 0.01

The final multiple linear regression models for predicting exam scores are shown in Table 4. Although a number of Anki user statistics were correlated with exam scores in the bivariate correlations, none were independently associated with exam scores for Course I, Course III, and the CBSE in the regression models. For Course II, the only statistically significant predictor was Anking % mature, which explained 36.2% of the variability in exam score. High dependency on Anki while studying was a significant predictor of Course I exam score and the CBSE. For Course III, the somewhat/fairly confidence level (compared to not at all/slightly) and the completely prepared level (compared to not at all/slightly) were both significant predictors of exam score.

**Table 4:** Final multiple linear regression models for associations between independent variables and exam scores

Exam and model variables	Beta coefficient (95% CI)	*p* value	*R*^2^	ANOVA *p* value
Course I (*n* = 48) MCAT percentile Anki dependency while studying = High (reference = Low)	0.2 (0.1–0.3) 4.6 (1.3–7.9)	< 0.001 0.008	45.9%	< 0.001
Course II (*n* = 35) AnKing % mature	0.8 (0.4–1.2)	< 0.001	36.2%	< 0.001
Course III (*n* = 23) How confident for exam = Somewhat/fairly (reference = Not at all/slightly) How prepared for exam = Completely (reference = Not at all/slightly)	6.7 (1.2–12.3) 11.3 (5.7–16.8)	0.020 < 0.001	49.2%	0.001
CBSE (*n* = 10) Anki dependency while studying = High (reference = Medium)	18.5 (0.6–36.4)	0.044	41.6%	0.044

*CBSE* Comprehensive Basic Science Exam, *CI* confidence interval

## Discussion

Medical students seek tools to help them build a strong foundation of knowledge and be successful with a multitude of in-house tests and national standardized examinations. Spaced repetition and retrieval-based practice are two strategies that assist with learning and long-term memory, and flashcards have been a mainstay tool for many years. Anki, an open-sourced software flashcard system, is continuing to evolve with feedback-rich statistics for the user as well as many choices of community developed flashcard decks. A very popular one is the AnKing deck, with over 30,000 flashcards covering almost all required medical school material. This was the most utilized deck by medical students. The second highest used platform, Physeo, a proprietary platform, is another with videos and image mnemonics specific for USMLE Step 1 and 2 with downloadable Anki cards based on their videos. Amboss™, another proprietary company, has also created a “tag” feature that highlights keywords in the Anki card that allows students to pull up specific Amboss content over such vocabulary. A variety of other open-platform in-house Anki decks shared among students were also used, although to a lesser extent. Finally, AnkiPalace and Ankihub are resources being utilized by medical students that simplify the configuration of Anki, diminish the learning curve necessary for day-to-day usage, and allow students to stay current on updates from the Anking deck. They have an integrated workshop with videos and instructions on how to properly utilize Anki in medical school [18, 19]. Future investigation should explore how students can best choose which decks will yield the most learning for the time and effort in a particular subject matter [23, 24].

Though medical students are using the Anki “system,” the literature on its effectiveness has been limited. In this study, we show that Anki usage in one first year medical school curriculum may be associated with increased test scores. There was a significant increase test scores on all course-specific summative examinations and the CBSE, even when accounting for MCAT scores. The CBSE, taken at the end of first year at BSOM, showed significantly higher scores in Anki users, with MCAT percentiles not differing between Anki and Anki non-users. This suggests that it benefits students with lower standardized test-taking skills, alongside being a free computer application. In addition, we found that the retention rate of Anki cards was significant for the CBSE. Since CBSE scores for Anki users had the largest differentiation compared to the course exams, we believe that long-term knowledge retention and integration of knowledge was achieved at a higher degree for Anki users. This demonstrates the effectiveness of Anki’s spaced repetition software, supporting the findings of this learning tool in the literature [17].

The exact mechanism by which Anki works is not consistent throughout the exams. For example, Course II final exam is an in-house exam that covers immunology, microbiology, and hematologic neoplasms, and related therapeutic agents and their mechanisms of action. It is a short 6-week course. Daily average of card numbers and scores were significantly correlated with test scores, much more so than the other two courses. It is possible that because Course II requires a great deal of memorization of new vocabulary, i.e., microorganisms, antibiotics, for which the flashcard construct combined with spaced repetition and retrieval-based practice is well-suited. The other two courses require learning new vocabulary, but there is more emphasis on principles and applying them to solving complex problems. Highlighting this, Course III performance on the NBME had the lowest correlation of daily average of cards. This module includes the integration of principles of cardiology, pulmonology, and nephrology, and less emphasis on memorization. For other examinations, we did not see a daily average and retention rate contribute significantly to exam scores. Interestingly, current streak and longest streak were significant for Course I but do not appear to influence the CBSE, suggesting that the usage of Anki every day may not matter. Rather, it may be how an individual is using the system. This metric is also limited due to the nature of missing one day eliminating the current streak. Additionally, the perceived notion of Anki dependency significantly influenced test scores in all modules except Staying Alive, suggesting that the more one uses spaced repetition, the more confident one becomes in taking an exam. Hence, although our findings indicate that Anki usage enhances performance in higher-level exams, we did not observe any consistent patterns in the platform’s utilization across all first-year exams. Consequently, it is advisable to adopt an individualized approach for using Anki to achieve higher exam scores until further research sheds light on these intricacies.

The modalities of Anki emphasize rote learning and recall of information. On the other hand, applying knowledge differs because it focuses on using this acquired information knowledge to solve problems, make decisions, or perform specific tasks. This requires a deeper understanding of concepts and their interconnectedness as well as how the learned information applies in specific contexts, such as diagnosing and treating patients in a clinical setting. Learning and memorization through Anki may not always facilitate the transfer of knowledge to new or unfamiliar situations, since applying learned knowledge involves the ability to transfer concepts to different scenarios. As such, one could hypothesize that courses that require greater memorization of knowledge versus application of knowledge may be better suited for Anki use [19, 25]. However, the current literature has limited information on which subjects Anki is most suitable for. Interestingly, recent publications have shown a correlation between USMLE Step 1 scores, but not USMLE Step 2 CK scores [17, 19, 20]. USMLE Step 1 emphasizes basic sciences, while USMLE Step 2 CK focuses on clinical application of medical knowledge [26]. Hence, it is plausible that Anki aids in the early development of medical knowledge but has limited applicability to direct clinical care.

This study has several limitations. It was conducted at a single US allopathic medical school, one with an integrated subject matter curriculum and all engaged/active learning strategies, e.g., no lectures, which limits the generalizability. However, we do consider our pilot program as a reproducible intervention to both “jump-start” students for using the Anki system and support their using the many valuable features. Although not directly surveyed, the peer-to-peer component could have potentially enhanced attendance for first year students. This idea is supported through methodologies that support peer-to-peer learning such as problem-based learning [27]. There could be a sample bias as students who found Anki useful or scored higher on examinations could have been more likely to respond to the surveys. These discrepancies have also been noted in other examples in the literature [18, 25]. For some of the analyses, sample sizes were small which limited the power of the study to find significant associations between Anki user statistics and exam scores. This was noted by the reduction in sample size from Course I (*n* = 48) to Course II (*n* = 39) to Course III (*n* = 23) to CBSE (*n* = 10). This was likely due to a combination of students not submitting surveys and some students choosing to not use Anki over time. Another limitation was the lack of randomization, which was not pursued given the ethical considerations behind limiting access to medical student resources. Thus, our findings could be a result of more motivated students using additional resources and spending more time studying, whether using Anki or not. We attempted to control for this using MCAT scores, but this confounder still exists. As new third-party resources continue to evolve in medical education, it will be imperative that future studies attempt to control for the multiple confounders that effect student performance. This will be the only way to truly comprehend the influence of these widely used resources in medical education. Finally, future studies evaluating Anki usage at multiple medical schools with larger sample sizes would determine whether the finding of higher academic performance with Anki usage is supported at other institutions.

## Conclusion

Spaced repetition techniques and retrieval-based practice are used extensively within the medical education community because of the enormous volume of information to be mastered. This study shows how Anki usage may be associated with an increased in standardized examination. The greatest benefits seen were with the CBSE, an approximation of USMLE Step 1. This supports Anki as an evidence-based spaced repetition and retrieval-based learning strategy for preparation of medical school summative and standardized examinations. We also found that Anki usage is significantly associated with increased exam scores regardless of a student’s inherent test-taking ability and may be beneficial for students with lower MCAT scores. Furthermore, it is free from financial burdens due to the system being free for computer usage. Additionally, we present a pilot program to facilitate early adoption of Anki that could be instituted at other schools to introduce the importance of the strategies to improve academic performance. However, our study found little correlation between its specific statistical markers and increased examination performance. Further research is needed to clarify how to specifically use Anki in order to optimize its benefits and whether the present study’s findings are generalizable to medical students at other schools. Our findings are highly relevant to both undergraduate and graduate medical education as Anki and other evidence-based learning tools may enhance long-term retention of medical knowledge, diminish time spent studying, and increase performance on standardized examinations. This is pertinent to physicians and medical students alike as the learning and preservation of biomedical knowledge is required for both board examinations and effective clinical care.

## Data Availability

The datasets generated during and/or analyzed during the current study are not publicly available due the data being stored by Boonshoft School of Medicine Department of Medical Education but may be available from the corresponding author on reasonable request.
